# Developmentally Regulated Rebound Depolarization Enhances Spike Timing Precision in Auditory Midbrain Neurons

**DOI:** 10.3389/fncel.2020.00236

**Published:** 2020-08-06

**Authors:** Hongyu Sun, Hui Zhang, Alysia Ross, Ting Ting Wang, Aycheh Al-Chami, Shu Hui Wu

**Affiliations:** Department of Neuroscience, Carleton University, Ottawa, ON, Canada

**Keywords:** auditory system, action potential, patch clamp recording, brain slice, calcium channel

## Abstract

The inferior colliculus (IC) is an auditory midbrain structure involved in processing biologically important temporal features of sounds. The responses of IC neurons to these temporal features reflect an interaction of synaptic inputs and neuronal biophysical properties. One striking biophysical property of IC neurons is the rebound depolarization produced following membrane hyperpolarization. To understand how the rebound depolarization is involved in spike timing, we made whole-cell patch clamp recordings from IC neurons in brain slices of P9–21 rats. We found that the percentage of rebound neurons was developmentally regulated. The precision of the timing of the first spike on the rebound increased when the neuron was repetitively injected with a depolarizing current following membrane hyperpolarization. The average jitter of the first spikes was only 0.5 ms. The selective T-type Ca^2+^ channel antagonist, mibefradil, significantly increased the jitter of the first spike of neurons in response to repetitive depolarization following membrane hyperpolarization. Furthermore, the rebound was potentiated by one to two preceding rebounds within a few hundred milliseconds. The first spike generated on the potentiated rebound was more precise than that on the non-potentiated rebound. With the addition of a calcium chelator, BAPTA, into the cell, the rebound potentiation no longer occurred, and the precision of the first spike on the rebound was not improved. These results suggest that the postinhibitory rebound mediated by T-type Ca^2+^ channel promotes spike timing precision in IC neurons. The rebound potentiation and precise spikes may be induced by increases in intracellular calcium levels.

## Introduction

Precise spike timing in auditory neurons permits them to encode important temporal features of sounds (Covey and Casseday, 1999; Oertel, 1999; Heil, 2004; Zheng and Escabi, 2013; Malone et al., 2015; Krächan et al., 2017; Runyan et al., 2017; Peterson and Heil, 2019). Certain neurons in the auditory lower brainstem have a remarkable capacity of firing that is precisely locked to a particular phase of a tone, a phenomenon known as phase locking (Oertel, 1999; Li et al., 2014; Peterson and Heil, 2019). These neurons have anatomical and biophysical specializations that enable them to fire action potentials with a precision of tens of microseconds (Oertel, 1997, 1999). In the auditory midbrain inferior colliculus (IC), neurons analyze and select temporal features of sounds. Many IC neurons are tuned to temporal parameters of sounds by integrating excitatory and inhibitory inputs in a special time window, creating selectivity to biologically important parameters of sounds, including sound duration, direction of frequency sweeps, amplitude and/or frequency modulation rate, and time intervals between sounds (Casseday et al., 2002; Jen et al., 2012; Macías et al., 2016; Ito, 2020). Temporal processing in the IC emphasizes aspects of temporal features that are relevant to biologically significant sounds. Intracellular studies of IC neurons *in vivo* show that many neurons respond to tones, frequency-modulated (FM) sweeps, and amplitude-modulated (AM) tones with leading inhibitory responses (Covey et al., 1996; Kuwada et al., 1997; Tan and Borst, 2007; Voytenko and Galazyuk, 2008; Geis and Borst, 2009, 2013; Valdizón-Rodríguez and Faure, 2017). Some neurons show an excitatory rebound after inhibition (Xie et al., 2008; Valdizón-Rodríguez and Faure, 2017). Extracellular recordings showed offset spikes of some IC neurons in response to ipsilateral tones (Zhang and Kelly, 2009; Lumani and Zhang, 2010). These firings following cessation of the tones are likely generated by postinhibitory rebound. The postinhibitory rebound has been proposed to play a critical role in the selection of sounds with special temporal features (Covey and Casseday, 1999; Nataraj and Wenstrup, 2005; Rajaram et al., 2019) and in processing binaural information (Zhang and Kelly, 2009; Lumani and Zhang, 2010).

Intracellular and whole-cell patch clamp recordings *in vitro* have shown that IC neurons display a rebound depolarization following membrane hyperpolarization (Smith, 1992; Sivaramakrishnan and Oliver, 2001; Ahuja and Wu, 2007; Sun and Wu, 2008a,b; Li et al., 2014). One or two spikes can be generated on a large rebound. The rebound depolarization not only affects membrane excitability but also considerably shortens the latency of the first spike when the neuron is subsequently depolarized (Sun and Wu, 2008a). However, the mechanisms as to how the rebound depolarization facilitates subsequent excitation and promotes spike timing are not known.

In the present study, we investigated how precise spike timing is enhanced by postinhibitory rebound and which ionic conductance is involved in the precise spike timing. By repeated hyperpolarization, we also examined whether the rebound could undergo a potentiation with improvement in the timing of the associated spikes.

## Materials and Methods

### Animals

Nine- to 21-day-old Wistar albino rats (male or female, Charles River, St. Constant, QC, Canada) were maintained in a 12-h light/dark cycle facility. All procedures in these experiments were performed in compliance with the guidelines of the Canadian Council on Animal Care and were approved by the Carleton University Animal Care Committee. Efforts were made to minimize animal suffering and the number of animals used.

### IC Slice Preparation

Experimental procedures are detailed in our previous reports (Sun and Wu, 2008a,b). Briefly, rat pups were decapitated rapidly under anesthesia with isoflurane. The brain was removed and dissected in 24–26°C artificial cerebral spinal fluid (ACSF), which was continuously bubbled with 95% O_2_ and 5% CO_2_. Brain slices were cut at 180–220 μm in the frontal plane through the auditory midbrain. The slice was then transferred to a recording chamber and completely submerged in the oxygenated ACSF, which circulated through the chamber at a flow rate of 2–2.5 ml/min with a temperature of 30–32°C. The ACSF contained (in mM) 129 NaCl, 3 KCl, 1.2 KH_2_PO_4_, 2.4 CaCl_2_, 1.3 MgSO_4_, 20 NaHCO_3_, 3 HEPES, and 10 glucose at a pH of 7.4 and had an osmolarity of 290–310 mOsm/kg. In some experiments, the T-type Ca^2+^ channel blocker mibefradil (5 μM), the specific h-channel blocker ZD7288 (10 μM), or the Na^+^ channel blocker tetrodotoxin (TTX, 1 μM) was added to the ACSF.

### Whole-Cell Patch Clamp Recording

Electrodes for whole-cell patch clamp recordings were prepared from thin-walled borosilicate glass pipettes. The electrode was filled with an internal solution containing (in mM) 130 K-gluconate, 0.6 EGTA, 10 HEPES, 2 MgCl_2_, 5 KCl, 2 ATP, and 0.3 GTP. The pH of the solution was adjusted to 7.25 with KOH, and the osmolarity was 280–290 mOsm/kg. The resistance of the patch electrode was 4–7 MΩ. In some experiments, the Ca^2+^ chelator 1,2-*bis*(2-aminophenoxy)ethane-*N*,*N*,*N*′,*N*′-tetraacetic acid tetrakis(acetoxymethyl ester; BAPTA AM) was added to the internal solution of the patch electrodes to replace EGTA. All chemicals and drugs were obtained from Sigma–Aldrich, St. Louis, MO, USA, except for BAPTA (Tocris, Ellisville, MO, USA).

**Figure 1:**
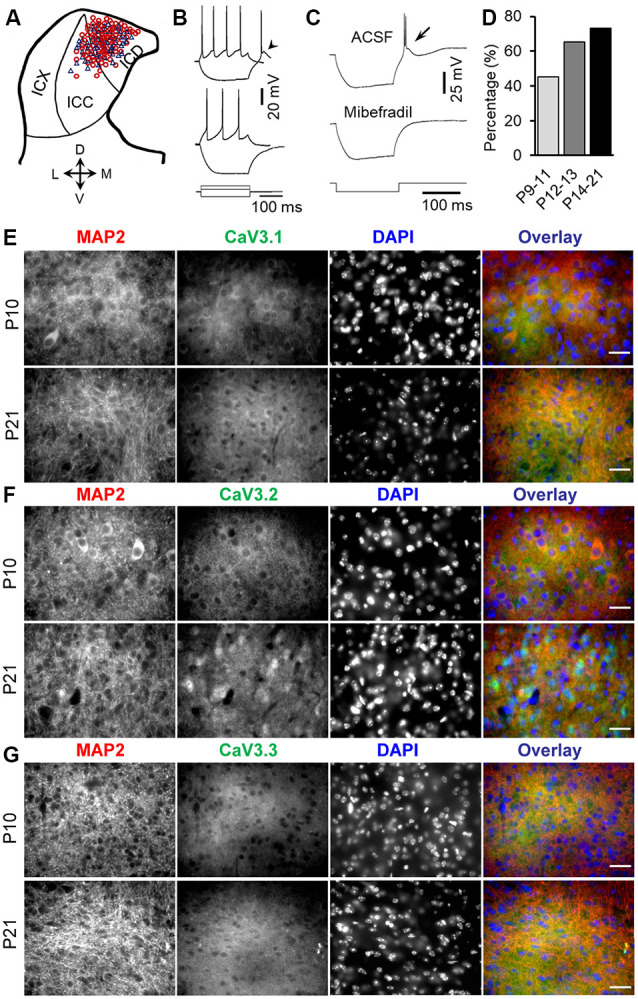
Developmental regulation of rebound depolarization in inferior colliculus (IC) neurons. **(A)** Distribution of rebound (red circle) and non-rebound (blue triangle) neurons in the IC. The IC was divided into the ICX (lateral region), ICC (middle region), and ICD (mediodorsal region). **(B)** Responses of a rebound neuron (top traces) and a non-rebound neuron (bottom traces) to a positive (60 pA) and a negative (−100 pA) current pulse. Arrowhead points to the rebound. **(C)** The rebound and anode break spikes (arrow) were blocked by 5 μm mibefradil. **(D)** Proportions of rebound neurons before (P9–11), during (P12–13), and after (P14–21) the onset of hearing. **(E–G)** Double-labeling immunocytochemistry with T-type calcium channel isoforms CaV3.1 (**E**, green), CaV3.2 (**F**, green), and CaV3.3 (**G**, green) and the microtubule-associated protein 2 (MAP2; **E**–**G**, red) in the IC from P10 and P21 rats demonstrates a developmental increase in the expression of CaV3.1, CaV3.2, and CaV3.3, with the highest changes in the CaV3.2 expression in IC neurons. Scale bars 20 μm.

Whole-cell patch clamp recordings were made by an EPC-8 patch-clamp amplifier (HEKA, Darmstadt, Germany) or Axon 200B amplifier (Axon Instruments, San Jose, CA, USA) from neurons located within the dorsal cortex (ICD) as well as the dorsal area of the central nucleus (ICC) of IC (Figure 1A). IC neurons were visualized under a Zeiss Axioskop microscope by a 40× water immersion objective with Hoffman modification contrast optics. Series resistances were in the range of 15–25 MΩ and were compensated by 50–70%. If the series resistance changed by more than 15% of the initial value during recording, the data were discarded. Signals were filtered at 5 kHz, digitized at 20 kHz by a Digidata 1320 interface, acquired and analyzed off-line by pCLAMP 8 (Axon Instruments, San Jose, CA, USA). All measurements were made from recordings at least 6–8 min after establishing a whole-cell configuration. Only neurons with stable resting potentials more negative than −55 mV were included for further analysis. The magnitude of the rebound was measured between the resting potential and the peak of the rebound. The first spike latency (FSL) was measured from the release of a hyperpolarizing current to the peak of the first spike. The depolarization rate of the first spike was determined by measuring the membrane potential slope in a 5–10 ms time window (d*v*/d*t*) right before the first spike initiation (Cudmore et al., 2010; Gastrein et al., 2011).

### Immunohistochemistry

Standard protocols were used for immunocytochemistry as described previously (Talos et al., 2006, 2012; Sun et al., 2013). Briefly, rat pups were transcardially perfused with 4% paraformaldehyde, and brainstems were dissected out and sectioned at 40 μm on a cryostat (Leica CM 3050S). Free floating IC sections from P10 and P21 rats were stained with rabbit anti-CaV3.1 (1:200, Alomone Labs, Jerusalem, Israel), rabbit Anti-CaV3.2 (1:200, Alomone Labs, Jerusalem, Israel), CaV3.3 (1:200, Alomone Labs, Jerusalem, Israel), and chicken anti-MAP2 (1:1,000, Novus Biologicals, Oakville, ON, Canada) antibodies. Fluorescent-conjugated secondary antibodies (Alexa Fluor 488 goat anti-rabbit, 1:1,000, and Alexa Fluor 647 goat anti-chicken, 1:1,000, Life Technologies Inc., Burlington, ON, USA) were used. False-positive staining was excluded by incubating control sections with only the secondary antibodies, adding no primary antibodies. Slides were examined with an epifluorescence microscope (Zeiss Axio Imager 2) with a fluorescence imaging digital camera and software.

### Statistical Analysis

Numerical averages are presented as means ± SEMs. Data were first tested for normality using the Shapiro–Wilk normality test. For normally distributed data, statistical significance was evaluated by two-tailed Student’s paired *t*-test, one-way ANOVA, or a repeated-measures one-way ANOVA with *post hoc* Student’s paired *t*-tests. The nonparametric two-tailed Mann–Whitney *U* test or a repeated-measures one-way ANOVA with *post hoc* Wilcoxon matched-pairs signed rank tests was used to evaluate non-normally distributed data. Correlations between the amplitude of rebound depolarization and the first spike jitter were determined using Pearson’s correlation test. The minimum criterion for statistical significance was set at *P* < 0.05.

## Results

### Developmental Regulation of Rebound Depolarization in IC

Whole-cell patch clamp recordings of 158 IC neurons from P9–21 rats were included in the present study (Figure 1A). The average resting potential was −59.9 ± 0.2 mV (*n* = 158). All of these neurons responded to the depolarizing current injection with sustained firing. One hundred two neurons (64.6%) showed a rebound depolarization accompanied by one or two action potentials following membrane hyperpolarization (Figure 1B, top traces). The remaining neurons did not have a rebound depolarization after hyperpolarization (Figure 1B, bottom traces). The rebound and non-rebound neurons were encountered in both ICC and ICD (Figure 1A). The rebound in IC neurons is mediated by low-threshold T-type Ca^2+^ channels as evidenced by the fact that a specific T-type Ca^2+^ channel blocker, mibefradil, completely blocked the rebound depolarization (Figure 1C). Interestingly, we found that the proportion of the rebound neurons in IC increased considerably from postnatal ages P9–11 (before the onset of hearing) to P14–21 (toward the maturity of auditory neurons; Figure 1D). The rebound was encountered in 19/42 (45.2%) neurons at P9–11 (18 rats), 15/23 (65.2%) neurons at P12–13 (15 rats), and 68/93 (73.1%) neurons at P14–21 (49 rats). However, the resting membrane potential and input resistance of neurons in these three age groups that can affect rebound generation were similar (P9–11: −59.4 ± 2.4 mV and 295.3 ± 13.3 MΩ; P12–13: 60.3 ± 0.45 mV and 285.6 ± 20.1 MΩ; P14–21: −59.6 ± 5.6 mV and 270.4 ± 14.3 MΩ, one-way ANOVA, *P* > 0.05 for both the resting potential and input resistance). Consistent with an increased proportion of rebound neurons in IC during development, we observed an overall increase in T-type CaV3.1, CaV3.2, and CaV3.3 (*n* = 4 for each group, Figures 1E–G) expression in IC neurons during development, with CaV3.2 showing the highest expression among these subtypes. In addition, CaV3.2 and MAP2 double labeling demonstrated CaV3.2 expression in cytoplasm and dendrites in IC neurons (Figure 1F). These data strongly support developmental regulation of rebound depolarization in IC neurons.

### Rebound Depolarization Promotes Spike Timing in IC Neurons

**Figure 2:**
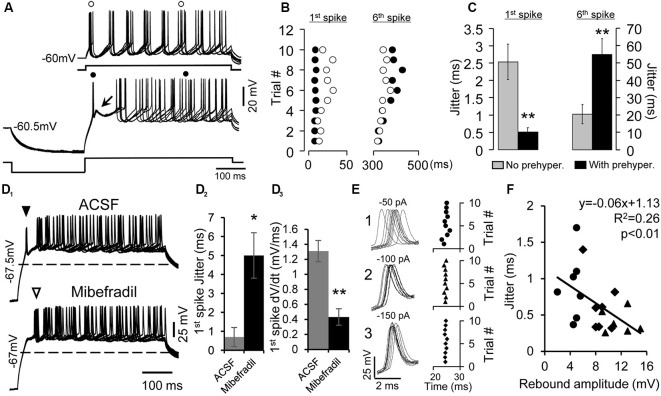
Enhancement of the precision of spike timing by rebound depolarization. **(A)** Overlapped traces of responses of a rebound neuron induced by five repetitive depolarizing pulses (50 pA, 500 ms) without (top traces) and with pre-hyperpolarizing (−100 pA, 250 ms, bottom traces) pulses. Arrow indicates the rebound. (o) and (•) indicate the first and sixth spikes without or with pre-hyperpolarization. **(B)** Raster plot of the first and sixth spikes elicited by depolarizing pulses with (•) and without (o) pre-hyperpolarization. Data are from the neuron shown in **(A)**. Each circle represents an action potential. **(C)** Mean jitter of the first and sixth spikes produced by depolarization with and without pre-hyperpolarization (*n* = 20). Error bars indicate SEM. Two-tailed Student’s paired *t*-test, **P < 0.01. **(D_1_)** Overlapped traces of responses in a rebound neuron induced by 10 depolarizing pulses (40 pA, 500 ms) with pre-hyperpolarizing pulses (−150 pA, 250 ms) in artificial cerebral spinal fluid (ACSF; top traces) and mibefradil (5 μM, bottom traces). Solid triangle points to first spikes that rode on the rebound. Open triangle indicates first spikes after the rebound was abolished. Dashed lines represent the resting membrane potential level. **(D_2_)** Mibefradil significantly increased jitter of the first spikes produced by depolarization with pre-hyperpolarization (*n* = 6). Error bars indicate SEM. Two-tailed Student’s paired *t*-test, *P < 0.05. **(D_3_)** Mibefradil significantly decreased the rate of membrane depolarization of the first spike (*n* = 6). Error bars indicate SEM. Two-tailed Student’s paired *t*-test, *P < 0.01. **(E)** Overlapped waveforms (left panel) and raster plot (right panel) of the first spikes induced by 10 depolarizing pulses (80 pA, 500 ms) with pre-hyperpolarizing pulses at three levels (−50, −100, and −150 pA). **(F)** Correlation of the rebound amplitude and spike jitter at pre-hyperpolarization of −50 pA (•), −100 pA (⧫), and −150 pA (▲, *n* = 7). Pearson’s correlation test, *P < 0.05.

We next investigated the precision of spike timing when the rebound neurons were repetitively depolarized with pre-hyperpolarization. The data presented below were all from rebound neurons. The neurons were depolarized with a fixed level of positive current injection for 10 times at 0.5 Hz (Figure 2A, top traces), and then they were hyperpolarized to a certain level before the same level of depolarizing current was applied again (Figure 2A, bottom traces). The time course and levels of current injection were chosen according to our previous studies (Sun and Wu, 2008b). The generation of rebound in IC neurons is time and voltage dependent. Different neurons required different levels and durations of hyperpolarization to generate maximum rebound. To elucidate how the rebound could enhance the precision of spike timing, in the present study, the magnitude and duration of positive or negative injected current were customized and held constant for each neuron. We set the hyperpolarizing current level between −50 and −200 pA for 100–250 ms and the depolarizing current level between +40 and +200 pA for 100–500 ms for different neurons. Current injection with these parameters could elicit a noticeable rebound and firing in our sampled cells, which would allow us to compare the precision of spike timing with or without pre-hyperpolarization within a neuron. We used spike jitter, which is defined as the standard deviation of 10 spike latencies, to quantify the spike precision. With pre-hyperpolarization, the precision of timing of the first spike was substantially improved (without pre-hyperpolarization, the jitter was 2.5 ± 0.5 ms; with pre-hyperpolarization, the jitter was 0.5 ± 0.1 ms; two-tailed Student’s paired *t*-test, *P* < 0.01, *n* = 20, Figures 2A–C). Interestingly, the precision of the later spikes was worse with pre-hyperpolarization than without pre-hyperpolarization (for example, without pre-hyperpolarization, the jitter of the sixth spikes was 20.5 ± 5.6 ms; with pre-hyperpolarization, the jitter of the sixth spikes was 54.9 ± 5.8 ms; two-tailed Student’s paired *t*-tests, *P* < 0.001, *n* = 20, Figures 2A–C).

Next, we verified the involvement of T-type Ca^2+^ channels in the precision of spike timing by blocking T-type Ca^2+^ channels with mibefradil (5 μM). We found that with mibefradil, the rebound and its associated spike were eliminated altogether (*n* = 11, Figure 2D_**1**_), and the new first spike was no longer precisely generated (Figure 2D_**2**_, open triangle, *n* = 6). The jitter of the 10 first-spike latencies was 0.7 ± 0.5 ms in ACSF and 5.0 ± 1.2 ms with mibefradil (*n* = 6, two-tailed Student’s paired *t*-test, *P* = 0.01). Fast membrane depolarization has been shown to promote spike timing precision in cortical neurons (Sourdet et al., 2003; Cudmore et al., 2010; Gastrein et al., 2011). We found that mibefradil significantly reduced the rate of membrane depolarization (1.31 ± 0.14 mV/ms in ACSF vs. 0.43 ± 0.11 mV/ms in mibefradil, *n* = 6, two-tailed Student’s paired *t*-test, *P* < 0.01, Figure 2D_**3**_).

Since generation of the rebound is voltage dependent (Sun and Wu, 2008b), i.e., an increase in the magnitude of pre-hyperpolarization leads to a larger rebound, we tested whether the precision of the first spike was also dependent on the level of pre-hyperpolarization (*n* = 7). Figure 2E (left traces) shows 10 waveforms of the first spikes generated by a fixed level of depolarizing current after the neuron was hyperpolarized with −50, −100, and −150 pA current. The current was applied 10 times at 0.5 Hz. The jitter of the spikes gradually decreased from 0.82 to 0.34 ms and again to 0.24 ms as the level of hyperpolarization was increased (Figure 2E, right panel). The magnitude of the rebound produced by hyperpolarization at the three levels was negatively correlated with the degree of spike jitter (*n* = 7, Pearson’s correlation test, *P* < 0.05, Figure 2F).

### Rebound Depolarization Is Modulated by Repetitive Hyperpolarizing Pulses

**Figure 3:**
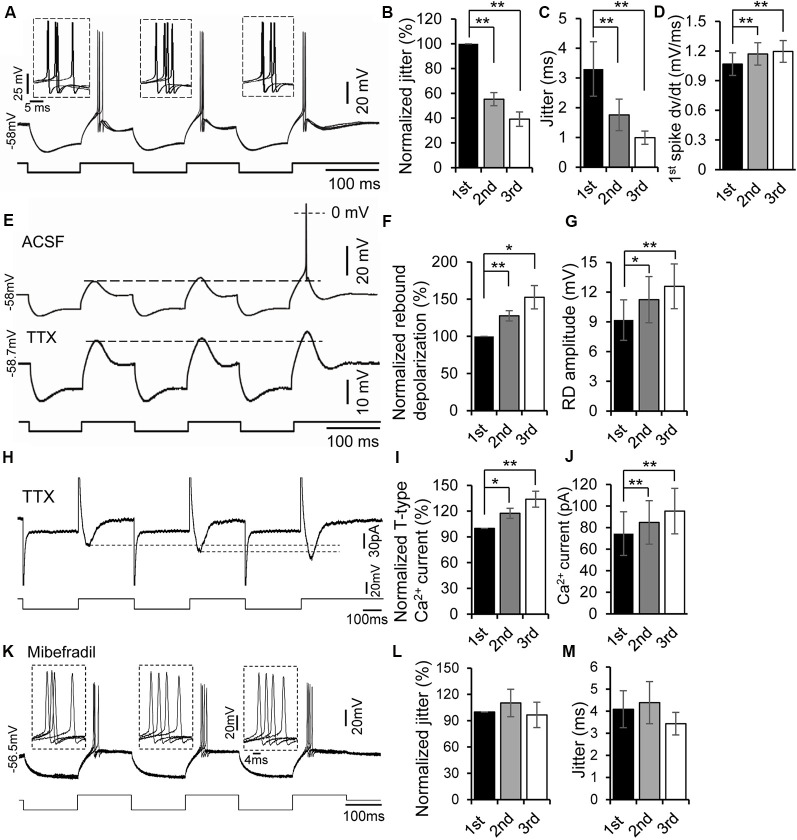
Rebound potentiation enhanced precision of spike timing. **(A)** Overlapped traces of responses in a rebound neuron induced by repetitive current injections of three hyperpolarizing pulses (−100 pA, 100 ms each) for four times at 0.5 Hz. Insets: overlapped waveforms of spikes on the first, second, and third rebounds. **(B,C)** Jitter **(C)** and normalized spike jitter **(B)** of the first spike produced by first, second, and third current pulses (*n* = 12). Error bars indicate SEM. *Post hoc* two-tailed Student’s paired *t*-test, **P < 0.01. **(D)** Depolarization rate (d*v*/d*t*) of the first spike produced by first, second, and third current pulses (*n* = 12). Error bars indicate SEM. *Post hoc* two-tailed Student’s paired *t*-test, **P < 0.01. **(E)** Responses of a rebound neuron to three repetitive hyperpolarizing pulses (−120 pA, 100 ms each) in ACSF (top trace, the spike was truncated) and in tetrodotoxin (TTX; bottom trace, dashed line indicates the peak of the first rebound). **(F,G)** Rebound depolarization amplitude **(G)** and normalized rebound depolarization **(F)** produced by first, second, and third current pulses (*n* = 8). Error bars indicate SEM. *Post hoc* two-tailed Student’s paired *t*-test, *P < 0.05, **P < 0.01. **(H)** Representative current responses of a rebound neuron to three repetitive hyperpolarizing voltage pulses in ACSF containing TTX (dashed line indicates the peak of the Ca^2+^ currents). **(I,J)** T-type Ca^2+^ current amplitude **(J)** and normalized rebound Ca^2+^ currents **(I)** evoked by first, second, and third voltage pulses (*n* = 5). Error bars indicate SEM. *Post hoc* two-tailed Student’s paired *t*-test, **P < 0.01. **(K)** Overlapped traces of responses in a rebound neuron induced by repetitive current injections of three hyperpolarizing pulses (−100 pA followed by +20 pA, 150 ms each) for four times at 0.5 Hz with mibefradil (5 μM). Insets: overlapped waveforms of spikes in response to the first, second, and third pulses. **(L,M)** Jitter **(M)** and normalized spike jitter **(L)** of the first spike produced by first, second, and third current pulses (*n* = 6). Error bars indicate SEM. Repeated-measures one-way ANOVA.

The rebound depolarization not only promoted the precision of spike timing but was itself potentiated when the rebound was produced by repetitive hyperpolarizing pulses within a few hundred milliseconds. Twelve neurons were tested by four trains of current injection at a frequency of 0.5 Hz; each train consisted of three repetitive 50–100 ms hyperpolarizing pulses at 5 Hz (for some neurons, each negative pulse was followed by a depolarizing pulse of 50–100 ms). With this protocol, the spikes on the later rebound were more precise and had a faster rate of membrane depolarization than those on the earlier rebound (Figures 3A–D). The second and third normalized spike jitters were 55.3 ± 5.3% (1.76 ± 0.53 ms, *n* = 12, *post hoc* two-tailed Student’s paired *t*-test, *P* < 0.01) and 39.2 ± 5.8% (0.996 ± 0.22 ms, *n* = 12, *post hoc* two-tailed Student’s paired *t*-test, *P* < 0.01) of the first one, respectively (3.30 ± 0.91 ms, *n* = 12, Figures 3B,C). The rates of membrane depolarization right before spikes evoked by second (1.171 ± 0.113 mV/ms, *n* = 12, *post hoc* two-tailed Student’s paired *t*-test, *P* < 0.01) and third pulses (1.196 ± 0.110 mV/ms, *n* = 12, *post hoc* two-tailed Student’s paired *t*-test, *P* < 0.01) were faster than those evoked by first pulses (1.068 ± 0.115 mV/ms, *n* = 12, Figure 3D). We then compared membrane hyperpolarizations induced by the three current pulses and found that the second and third membrane hyperpolarizations were 100.3 ± 1.9% and 98.9 ± 2.3% of the first one (*n* = 12, repeated-measures one-way ANOVA, *P* > 0.05). Thus, the possibility that the increased precision of the later spikes and larger rebound might be attributed to larger membrane hyperpolarization could be ruled out. Importantly, application of the T-type Ca^2+^ channel blocker mibefradil (5 μM) abolished the enhancement of spike precision induced by three repetitive hyperpolarizing pulses in rebound IC neurons (Figures 3K–M), confirming the involvement of T-type Ca^2+^ channels. The second and third normalized spike jitters were 110.26 ± 15.65% (4.38 ± 0.84 ms, *n* = 6, repeated-measures one-way ANOVA, *P* > 0.05) and 96.62 ± 14.50% (3.43 ± 0.51 ms, *n* = 6, repeated-measures one-way ANOVA, *P* > 0.05) of the first one, respectively (4.09 ± 0.84 ms, *n* = 6, Figures 3L,M).

**Figure 4:**
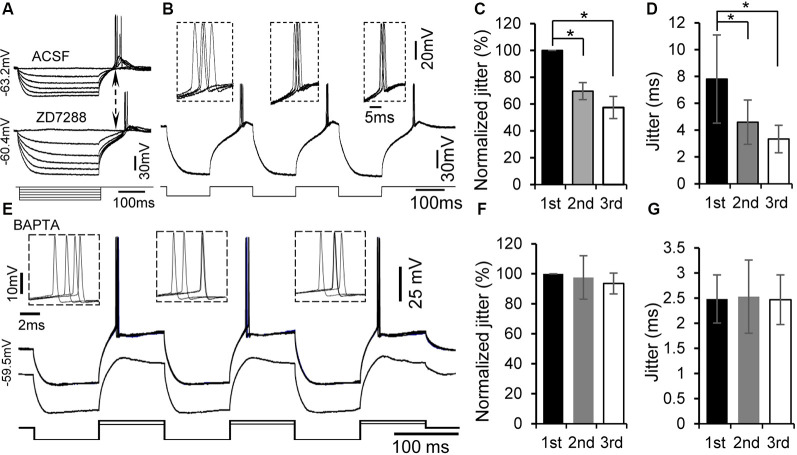
Rebound potentiation and enhancement of spike timing were intracellular Ca^2+^ dependent but h-channel independent. **(A)** Representative voltage responses to hyperpolarizing current injections with or without the specific h-channel blocker ZD7288 (10 μM). **(B)** Overlapped traces of responses in a rebound neuron induced by repetitive current injections of three hyperpolarizing pulses for four times at 0.5 Hz with ZD7288 (10 μM). Insets: overlapped waveforms of spikes in response to the first, second, and third pulses. **(C,D)** Jitter **(D)** and normalized spike jitter **(C)** of the first spike produced by first, second, and third current pulses (*n* = 6). Error bars indicate SEM. *Post hoc* two-tailed Student’s paired *t*-test **(C)** or Wilcoxon matched-pairs signed rank test **(D)**, *P < 0.05. **(E)** With 5 mM BAPTA in the internal solution of the electrode, overlapped traces of responses to three repetitive hyperpolarizing pulses (−180 pA, 100 ms), each of which was followed by a depolarizing pulse (120 pA, 100 ms), for four times at 0.5 Hz (top trace). Responses to three repetitive hyperpolarizing pulses (−180 pA, 100 ms), each of which was followed by a depolarizing pulse (60 pA, 100 ms; bottom trace). Note that the rebound was not potentiated by repetitive hyperpolarization. Insets: overlapped waveforms of spikes to first, second, and third depolarizing pulses. **(F,G)** Jitter **(G)** and normalized jitter **(F)** of spikes elicited by first, second, and third depolarizing pulses following hyperpolarizing pulses with 5 mM BAPTA in the internal solution (*n* = 6). Error bars indicate SEM. Repeated-measures one-way ANOVA.

In the next set of experiments, we examined whether the preceding rebound was necessary to induce rebound potentiation. When membrane hyperpolarization was too small to produce a rebound by the first current pulse, no rebound was generated by either the second or third pulse. However, once the hyperpolarizing current level was large enough to generate a rebound (i.e., the first rebound), second and third rebounds were always produced. A spike was often generated on the third rebound (Figure 3E, top trace). To demonstrate further if the later rebound was larger than the previous one, we applied TTX (1 μM) to block the spikes (*n* = 8) and then measured the magnitude of each rebound. With TTX in ACSF, a progressive increase in magnitude of the rebounds within a series was evident (Figure 3E, bottom trace). The second and third rebound magnitudes were 127.6 ± 6.9% (11.24 ± 2.33 mV, *n* = 8, *post hoc* two-tailed Student’s paired *t*-test, *P* < 0.01) and 152.6 ± 15.7% (12.59 ± 2.26 mV, *n* = 8, *post hoc* two-tailed Student’s paired *t*-test, *P* < 0.01) of the first one (9.18 ± 2.04 mV, *n* = 8, Figures 3F,G), respectively. To validate further the potentiation of low-threshold T-type Ca^2+^ currents in IC neurons, we measured the amplitudes of T-type Ca^2+^ currents evoked by three repetitive hyperpolarizing pulses under the voltage clamp mode. Similar to the current clamp findings, we found a progressive increase in the magnitude of T-type Ca^2+^ currents within a series of repetitive pulses (Figures 3H,J). The second and third T-type Ca^2+^ current amplitudes were 117.51 ± 3.97% (84.82 ± 20.16 pA, *n* = 5, *post hoc* two-tailed Student’s paired *t*-test, *P* < 0.05) and 133.99 ± 7.38% (95.34 ± 21.10 pA, *n* = 5, *post hoc* two-tailed Student’s paired *t*-test, *P* < 0.01) of the first one (74.48 ± 20.26 pA, *n* = 5, Figures 3I,J), respectively. All these results suggest that the rebound potentiation was likely induced by the previous rebound depolarization mediated by T-type Ca^2+^ channels.

h-Channels are prominent in many IC neurons and known to interact with T-type Ca^2+^ channels (Sun and Wu, 2008b; Engbers et al., 2013; Cazade et al., 2017). We therefore determined whether h-channel activity regulates rebound potentiation in IC neurons (*n* = 6). Consistent with our previous study (Sun and Wu, 2008b), application of h-channel blocker ZD7288 (10 mM) did not prevent the generation of rebound depolarization and spiking (Figure 4A). Moreover, ZD7288 did not significantly suppress the rebound potentiation and associated enhancement of spike precision induced by three repetitive hyperpolarizing pulses in IC neurons (Figures 4B–D). The second and third normalized spike jitters were 69.54 ± 6.33% (4.59 ± 1.65 ms, *n* = 6, *post hoc* two-tailed Student’s paired *t*-test, *P* < 0.01) and 57.45 ± 8.27% (3.34 ± 1.03 ms, *n* = 6, *post hoc* two-tailed Student’s paired *t*-test, *P* < 0.01) of the first one (7.81 ± 3.29 ms, *n* = 6, Figures 4C,D), respectively.

If generation of the rebound leads to potentiation of the subsequent rebound, one possible mechanism for the rebound potentiation might be an increase in intracellular Ca^2+^ levels. We tested this hypothesis by recording with patch electrode containing a fast Ca^2+^ chelator, BAPTA (5 mM), in the internal solution (*n* = 7). A similar protocol to that for examining rebound potentiation was applied to these neurons. The rebound potentiation could still be seen at the early time of the recording. About 20–30 min after establishing the whole-cell configuration, neither rebound potentiation (Figure 4E, bottom trace) nor reduction in spike jitter was observed (Figure 4E, top trace). The second and third spike jitters were 98.43 ± 14.42% (2.53 ± 0.73 ms, *n* = 7, repeated-measures one-way ANOVA, *P* > 0.05) and 96.50 ± 7.61% (2.47 ± 0.49 ms, *n* = 7, repeated-measures one-way ANOVA, *P* > 0.05) of the first spike jitter (2.48 ± 0.48 ms, *n* = 7, Figures 4F,G), respectively. These data support a calcium-dependent mechanism mediating the rebound potentiation in IC neurons.

## Discussion

IC neurons are critically involved in processing important temporal features of sounds (Wenstrup et al., 2012; Chen et al., 2019; Yang et al., 2020). One striking biophysical property of IC neurons is rebound depolarization (Sun and Wu, 2008a; Kasai et al., 2012; Chandrasekaran et al., 2013; Ayala and Malmierca, 2018). However, how the rebound depolarization affects spike timing of IC neurons is unknown. The present study reveals that T-type Ca^2+^ channel-mediated rebound depolarization was developmentally regulated in IC neurons. Rebound depolarization significantly increased the spike timing precision in IC neurons. Interestingly, the rebound depolarization itself could be potentiated following one or two preceding rebounds through a calcium-dependent mechanism. Therefore, we demonstrate for the first time that rebound depolarization can enhance the temporal precision of spike timing in IC neurons during development.

The proportion of rebound neurons increased from less than 50% before hearing onset to more than 70% after hearing onset, i.e., 2–3 weeks after birth. Because generation of the rebound is voltage dependent (Sun and Wu, 2008b), the incidence of rebound neurons might have been influenced by a neuron’s resting membrane potential and input resistance. But our results showed that IC neurons did not undergo significant changes in the resting membrane potential or input resistance during the period of P9–21. However, the proportion of IC neurons with rebound depolarization continued to increase after the onset of hearing. The increased number of rebound neurons in the IC during development was further confirmed by increased expression of low-threshold T-type Ca^2+^ channels with age (Figure 1; Yunker et al., 2003). These data strongly suggest a potential role of rebound depolarization in auditory processing as an animal’s hearing matures.

An important novel finding of the present study was the significant enhancement in precision of first-spike latency when the neuron was repetitively depolarized following hyperpolarization at a low rate (e.g., 0.5 Hz). The spike jitter was only 0.5 ms, which was five times smaller than that of the first spike produced by the same level of depolarization without pre-hyperpolarization. These results suggest that activation of T-type Ca^2+^ channels promotes generation of the early spike and enhances its precision. T-type Ca^2+^ channels in IC neurons can be activated at −60 mV after removing its inactivation, i.e., deinactivation, by hyperpolarization (N’Gouemo and Rittenhouse, 2000; N’Gouemo and Morad, 2003). Depolarizing current following hyperpolarization may deinactivate and then activate T-type Ca^2+^ channels, producing a fast-rising rebound depolarization and precise spiking. The process of the deinactivation is voltage dependent. Larger hyperpolarization would deinactivate more channels, producing a larger rebound (Perez-Reyes, 2003; Alexander et al., 2006; Weiss and Zamponi, 2019) and therefore more precise spiking (Figure 2). The negative correlation between the magnitude of the rebound and spike jitter further supports this idea (Figure 2). Consistent with previous studies (Sourdet et al., 2003; Cudmore et al., 2010; Gastrein et al., 2011), enhancement of spike timing precision is associated with a faster rate of membrane depolarization right before the spike initiation.

Interestingly, the rebound was potentiated following one or two preceding rebounds within only a few hundred milliseconds. Similar potentiation of T-type Ca^2+^ currents was confirmed using voltage clamp recordings. The spike that rode on the potentiated rebound became more precise. We did not systematically investigate the temporal limits of the repetitive hyperpolarization pulse required for inducing rebound potentiation. Nevertheless, the results suggest that a relatively short (less than 100 ms) but successive membrane hyperpolarization, e.g., synaptic inhibition (GABAergic or glycinergic postsynaptic potentials) or after-hyperpolarization (AHP) of a train of action potentials, in IC neurons can easily generate potentiated rebound and thereby precise spiking. Our results reveal that the potentiation is Ca^2+^-dependent because the rebound potentiation was eliminated with BAPTA, a fast-acting Ca^2+^ chelator that would be expected to reduce intracellular Ca^2+^ within a few milliseconds (Ouanounou et al., 1999; Cazade et al., 2017; Chemin et al., 2019). Generation of the rebound in neurons of some other brain areas has been attributed to hyperpolarization-activated h-currents (Aizenman and Linden, 1999; Koch and Grothe, 2003; Surges et al., 2006; Engbers et al., 2013; Cazade et al., 2017). Although we observed the sag of membrane potential when the neuron was hyperpolarized, which reflected activation of h-channels, generation of the rebound in IC neurons does not rely on activation of h-channels (Sun and Wu, 2008b). Importantly, blockade of h-channels did not suppress the rebound potentiation and associated enhancement of spike precision, ruling out the possible involvement of h-channels. Our findings are generally consistent with previous studies in thalamic neurons showing potentiation of T-type Ca^2+^ current by pre-hyperpolarization for around 1 s followed by a brief depolarization (Leresche et al., 2004; Bessaïh et al., 2008). However, our protocol of current injection consisted of much shorter hyperpolarizing pulses at a higher frequency. The potentiation of T-type current in IC neurons seemed to occur faster than in thalamic neurons. Specific isoforms of T-type Ca^2+^ channels encoded by different genes (i.e., CaV3.1, CaV3.2, and CaV3.3) show different activation and inactivation kinetic features (Murbartián et al., 2004; Cazade et al., 2017; Chemin et al., 2017, 2019). Using tsA-201 cells expressing different isotypes of T-type Ca^2+^ channels, Chemin’s group has recently shown isotype-specific regulation of T-type Ca^2+^ channels by Ca^2+^ ions (Cazade et al., 2017; Chemin et al., 2017, 2019). A rise of intracellular Ca^2+^ can inhibit CaV3.3 currents through a phosphorylation mechanism while Ca^2+^ ions can induce small enhancement in CaV3.2 currents (Cazade et al., 2017). Our immunohistochemistry results show much stronger expression of CaV3.2 than CaV3.1 and CaV3.3 in developing IC neurons supporting the fact that distinct isotypes of T-type Ca^2+^ channels in IC neurons may attribute to a fast potentiation of the rebound. Further studies will be needed to understand the underlying mechanism of the rebound depolarization modulation in IC neurons.

Many IC neurons respond to tones, FM sweeps, and AM tones with an early inhibitory synaptic potential (IPSP) followed by robust offset firing (Covey et al., 1996; Kuwada et al., 1997; Tan and Borst, 2007; Voytenko and Galazyuk, 2008; Geis and Borst, 2009, 2013; Kuo and Wu, 2012; Lee et al., 2019). Some of these neurons show selectivity to tone duration, FM or AM rate, and the delay between two tones. A number of hypotheses and theoretical models have attempted to explain the neuronal selectivity to these temporal features of sounds (Ehrlich et al., 1997; Covey and Casseday, 1999; Borisyuk et al., 2002; Large and Crawford, 2002; Nataraj and Wenstrup, 2005; Yin et al., 2008; Kuo and Wu, 2012; Williams and Fuzessery, 2012; Lee et al., 2019). Prominent inhibitory responses have been reported to present in dorsal IC neurons in awake animals *in vivo* (Xie et al., 2007; Wong and Borst, 2019) and are often associated with sound offset responses that have an essential role in duration encoding and discrimination (Sayegh et al., 2011; Kopp-Scheinpflug et al., 2018). Postinhibitory rebound is a possible key component involved in processing these offset spiking features observed *in vivo* by providing temporally precise offset excitation following inhibition (Pollak et al., 2011; Kasai et al., 2012; Wong and Borst, 2019). This is in agreement with recent studies showing that communication calls induce a summation of synaptic inhibition at various frequency bands and trigger prominent offset responses (Sanchez et al., 2008; Akimov et al., 2017). The concept is that IC neurons respond to a favored temporal feature by increasing the probability of offset firing due to coincidence of the postinhibitory rebound with an excitatory input. Our results support these hypotheses but also provide the first biophysical evidence for enhancement of precise spiking by activation of T-type Ca^2+^ channels when a neuron receives early inhibition. Precise spike timing is critical for sound temporal coding in auditory neurons (Covey and Casseday, 1999; Oertel, 1999; Heil, 2004; Zheng and Escabi, 2013; Malone et al., 2015; Krächan et al., 2017; Runyan et al., 2017; Peterson and Heil, 2019). Neurons in ICD and dorsal ICC are heavily innervated by a combination of ascending and descending auditory inputs, as well as nonauditory inputs (Chen and Song, 2019; Wong and Borst, 2019; Yang et al., 2020), and are involved in processing complex multimodal information with temporal features (Wong and Borst, 2019). Therefore, the millisecond-precise first-spike timing may enable sufficiently rapid encoding of stimulation information and offer a fast and reliable mechanism for processing specific temporal features of acoustic information in the IC.

## Data Availability Statement

All datasets generated for this study are included in the article.

## Ethics Statement

The animal study was reviewed and approved by the Carleton University Animal Care Committee.

## Author Contributions

HS and SW designed the study. HS, HZ, AR, TW, and AA-C conducted the experiments. HS, HZ, AR, TW, and AA-C analyzed the data. HS, HZ, AR, TW, AA-C, and SW wrote the manuscript.

## Conflict of Interest

The authors declare that the research was conducted in the absence of any commercial or financial relationships that could be construed as a potential conflict of interest.
